# Variation and Correlation Analysis of Flavour and Bacterial Diversity of Low-Salt Hotpot Sauce during Storage

**DOI:** 10.3390/foods12020333

**Published:** 2023-01-10

**Authors:** Yanan Xia, Bayaer Eerdun, Junlin Wang, Yankai Li, Quan Shuang, Yongfu Chen

**Affiliations:** 1 Key Laboratory of Dairy Biotechnology and Engineering, Inner Mongolia Agricultural University, Ministry of Education, Hohhot 010018, China; 2 College of Food Science and Engineering, Inner Mongolia Agricultural University, Hohhot 010018, China; 3 Inner Mongolia Red Sun Food Co., Ltd., Hohhot 010020, China

**Keywords:** hotpot sauce, low-salt, flavour, taste, bacterial composition

## Abstract

Culinary circles have experienced a recent trend towards low-salt hotpot sauces. Here, changes in the physicochemical quality, flavour, and bacterial diversity of hotpot sauces with different salt concentrations were studied during storage. The results indicated that the peroxide and acid values of hotpot sauce increased gradually and that the quality began to deteriorate with storage. A storage temperature of 4 °C and salt concentration above 4.4% significantly reduced spoilage. The salt concentration had no significant effect on the flavour but extended storage resulted in significant differences in flavour reflected in the changes of sweet, sour, bitter, umami, aftertaste-A, abundance, organic sulphide, and alkanes. Significant differences were found in the bacterial composition between samples stored at different temperatures. *Norank-f-o-Chloroplast* was the main bacterium in the samples stored at low temperatures, which was beneficial for preservation. *Bacillus* was detected in 4.1% NaCl samples stored at 25 °C, directly promoting sauce spoilage and an unpleasant flavour. This bacterium signalled the spoilage of low-salt hotpot sauce stored at room temperature.

## 1. Introduction

Hyperlipidaemia and hypertension are major public health issues; the prevalence of hypertension among adults in China has been reported to be as high as 25% [1]. The likelihood of hypertension increases with a high salt intake [2]. Thus, an appropriate salt intake is necessary for the maintenance of health.

Hotpot has long been consumed in China and is increasing in popularity worldwide. Hotpot sauce is produced from peanut kernels, sesame, leek sauce, beans, garlic, sweet bean sauce, salt, sugar, spices, etc. It is rich in protein, fat, vitamins, and nutrients [3,4]. However, the complexity of ingredients causes hotpot sauce to be vulnerable to microbial contamination and spoilage and long-term storage may lead to adverse changes in quality, such as nutrient degradation, rancidity, browning, and flavour degradation. The salt content of hotpot sauces on the market can be as high as 6%. Although this meets the requirement of less than 25% salt content in the enterprise standard for hotpot sauces (Q/LXAD0004S-2017), it is still inappropriate from the perspective of people’s health because it is significantly higher than the recommended salt intake of 6 g [5,6]. Therefore, it is necessary to develop low-salt hotpot sauce for health purposes.

The research on hotpot sauce is mainly concerned with formula development and the use of poppy shells [7,8] and Sudan dyes [9]. There has been little research on low-salt hotpot sauce and storage quality. Gu et al. [10] studied quality changes in beef tallow hotpot soup bases under conditions of cold storage (4 °C) and reported that the moisture, acid values, and peroxide values (AV and POV, respectively) of samples all increased over time, along with the oxidation rate. The unsaturated fatty acid content decreased and the saturated fatty acid content increased during storage. Therefore, to maintain product quality and prolong shelf life, it is necessary to control the deterioration of oils and fats [10]. However, this is not sufficient. Low-salt sauces may not only exacerbate the deterioration of fatty acids but also affect the taste of products and promote the growth of spoilage-related microorganisms during storage [11].

At the same time, microorganisms are involved in the natural storage process of hotpot sauce; some corrupt microorganisms cause sample corruption and deterioration, but specific microorganisms can decompose large molecular substances in the dipping, further forming small molecular flavour substances to promote the flavour of the sauce. For Chinese fish sauce (Yu-lu), five microbial genera—*Halanaerobium*, *Halomonas*, *Tetragenococcus*, *Halococcus*, and *Candidatus Frackibacter*—constitute the core microbial flora responsible for flavour formation [12,13]. The microbial metabolic pathways degraded raw materials into primary metabolites, such as glucose, amino acids, and fatty acids [12,13]. Meanwhile, *H. erinaceus* significantly promoted the accumulation of esters and alcohols and positively influenced the sensory constituents [12,13]. *Bacillus*, *Staphylococcus*, and *Psychrobacter* positively correlated with flavour formation with the addition of *H. erinaceus* [12,13]. Therefore, it is necessary to study the microbial composition of hotpot sauce and its effect on flavour and quality during storage.

This study was performed to examine changes in the AV and POV of low-salt hotpot sauce during storage, analyse the influence of salt concentration on flavour and volatile flavour compounds, further clarify the influence of low salt concentrations on the bacterial composition of hotpot sauce, and provide a theoretical basis for the development and quality optimization of low-salt hotpot sauce.

## 2. Materials and Methods

### 2.1. Samples

**Figure 1:**
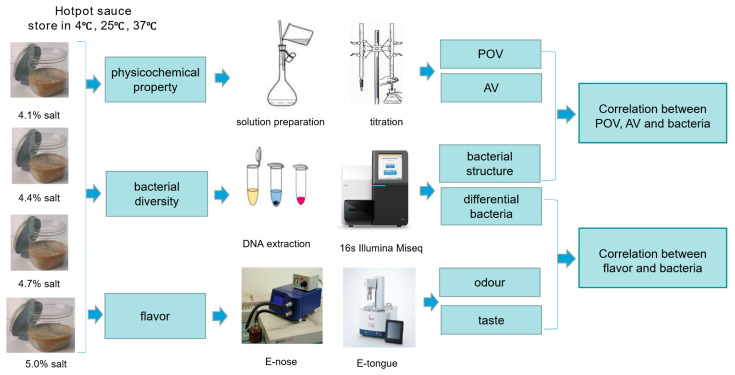
The whole research process.

Low-salt hotpot sauces (salt concentrations of 4.1%, 4.4%, 4.7%, and 5.0%) were provided by the Inner Mongolia Red Sun Food Co., Ltd. (Hohhot, Inner Mongolia, China). The hotpot dipping was produced with peanut kernel, sesame, salted leek sauce, soybean, garlic, sweet sauce, edible salt, sugar, and spices as raw materials, according to a certain proportion of mixed frying. The samples were stored at 4 °C, 25 °C, and 37 °C without additives, such as preservatives, and were sampled every month (0, 1, 2, and 3 month) for analysis. The test process is shown in Figure 1.

### 2.2. Determination of Peroxide Value (POV)

The samples were loaded into a Soxhlet fat extraction device. Then, 2–3 times the sample volume of petroleum ether was added and heated and reflux extraction was performed for 12 h. All the extracts were collected and combined in a flask and placed in a rotary evaporator with a water bath temperature of 40 °C. The solvent was thoroughly removed under a negative pressure of 0.08–0.1 Mpa. The remaining liquid oil was used as the sample to determine the acid value (AV) and POV [14].

The liquid oil was weighed (m) and placed in a 250 mL iodine flask, to which 30 mL of a trichloromethane–glacial acetic acid mixture was added, followed by gentle shaking to completely dissolve the sample. Then, 1 mL of saturated potassium iodide solution was added to the flask and the cap was closed. The flask was shaken gently for 0.5 min and placed in the dark for 3 min. Then, 100 mL of water was added, the flask was shaken well, and the filtrate was immediately titrated. The iodine was precipitated with 0.002 mol/L sodium thiosulphate standard solution. Once the mixture had turned light yellow, 1 mL of starch indicator was added, the titration was continued, and the samples were shaken until all blue colour had disappeared from the solution (V). A blank was tested simultaneously. The volume (V0) of 0.01 mol/L sodium thiosulphate solution consumed in the blank test did not exceed 0.1 mL. The peroxide was calculated using the following Formula (1):

(1) X_POV_ = (V − V0) × 0.002 × 0.1269 × 100/m

### 2.3. Determination of Acid Value (AV)

The liquid oil was weighed (m) and 70 mL of ether–isopropyl alcohol mixture was added, along with four drops of phenolphthalein indicator, and shaken vigorously. Then, 0.5 mol/L NaOH was used for titration. The titration endpoint was when the sample solution initially appeared reddish and there was no obvious fading within 15 s. The volume (V) of the standard titration solution consumed during titration was recorded. The same volume of an ether–isopropyl alcohol mixture and phenolphthalein indicator was added to another 250 mL conical flask. The mixture was vortex-mixed and titrated to the endpoint of 0.5 mol/L NaOH. The volume (V0) consumed was recorded [14]. The AV was calculated according to the following Formula (2):

(2) X_AV_ = (V − V0) × 0.5 × 56.1/m

### 2.4. Taste Quality Evaluation of Hotpot Sauce by E-Tongue

A total of 70 mL of hot water was added to samples of 35 g and centrifuged at 3000× *g* to obtain the supernatant. The supernatant was kept at 4 °C for 12 h to remove the top oil and obtain the treated samples. Then, 80 mL of the treated sample was placed in the plastic cup of an electronic tongue (SA402B, Insent, Beijing Ensoul Technology Ltd., Beijing, China). The probe was cleaned with pure water before each measurement. The voltage was from −1 to 1 V, the pulse interval was 100 mV, and the sensitivity was 10^5^ [15]. Based on the bionic principle, the electronic tongue simulates the human taste system to recognize different tastes in food (bitterness, astringence, sweetness, sourness, umami, saltiness, aftertaste A, aftertaste B, and richness). PCA, CA, and other statistical analysis methods can be used to analyse the differences in various tastes in different samples.

### 2.5. Flavour Quality Evaluation of Hotpot Sauce by E-Nose

Then, 7–8 mL samples were added to test tubes, sealed with plastic wrap, placed in a water bath at 70 °C for 30 min, and examined using the PEN3 electronic nose (German AIRSENCE company, Schwerin, German). The detection, cleaning, and pre-sampling times were 120 s, 80 s, and 5 s, respectively. Both the sample and carrier gas flow rates were 400 mL/min. At the beginning of the measurement, the sensor started to fluctuate with time and flattened when it reached 110 s. The data were obtained at 118, 119, and 120 s for analysis and the different groups of samples were tested in parallel [16]. The electronic nose uses the response pattern of the gas sensor array to recognize flavour based on the bionic mechanism. The 10 sensors had strong response values to different types of compounds (Table 1) and multivariate statistical methods (PCA, CA, PLS-DA, etc.) could also be used to compare the similarity and difference of volatile flavour in different groups of samples.

### 2.6. Total DNA Extraction, PCR Amplification, and MiSeq High-Throughput Sequencing

The total DNA was extracted from the microbial community using an E.Z.N.A.^®^ soil DNA Kit [17] (Hangzhou Xidon Biotechnology Co., Ltd., Hangzhou, China). After successful extraction, the quality of the DNA was determined using 1% agarose gel electrophoresis and the concentration and purity of DNA were determined using spectrophotometry (NanoDrop2000; China Thermo Fisher Scientific Ltd., Shanghai, China).

With reference to the method of Xia et al. [18], the V3–V4 variable region of the bacterial 16S rRNA gene was amplified by PCR (upstream primer: 338F (5′-ACTCCTACGGGAGgCAGCAGg-3′); downstream primer: 806R (5′-GGACTachVGGGTWTCTAat-3′). The PCR profile consisted of a pre-denaturation step at 95 °C for 3 min followed by 27 cycles of denaturation at 95 °C for 30 s, annealing at 55 °C for 30 s, extension at 72 °C for 30 s, and a final extension at 72 °C for 10 min, followed by storage at 4 °C.

The PCR mix consisted of 4 μL of 5× TransStart FastPfu buffer, 2 μL of dNTP solution (2.5 mmol/L), 0.8 μL of upstream primer (5 μmol/L), 0.8 μL of downstream primer (5 μmol/L), 0.4 μL of TransStart FastPfu DNA polymerase (Beijing TransGen Biotechnology Co., Ltd., Beijing, China), and 10 ng of template DNA comprising 20 μL of distilled water. Each sample was analysed in three replicates and the PCR products of the replicates were mixed and detected using 2% agarose gel electrophoresis. The PCR products were purified with an AxyPrep DNA Gel Extraction Kit (Shanghai Jinpan Biotechnology Co., Ltd., Shanghai, China) and then detected and quantified using fluorometry (Quantus™, Promega Corporation, Fitchburg, WI, USA). The mixing proportions were in accordance with the sequencing quantity requirements of each sample. The samples were subjected to high-throughput sequencing on the Illumina MiseqPE300 platform by Shanghai Majorbio Technology Co., Ltd., Shanghai, China.

### 2.7. Data Processing and Bioinformatics Analysis

Trimmomatic software (http://www.usadellab.org/cms/index.php?page=trimmomatic (accessed on 1 August 2014), Bolger Anthony M, Lohse Marc, and Usadel Bjoern, Max Planck Institute of Molecular Plant Physiology, Jülich, Germany) was used for quality control of the original sequencing results. FLASH software (http://ccb.jhu.edu/software/FLASH/index.shtml (accessed on 7 September 2011), Magoč Tanja, and Salzberg Steven L, Johns Hopkins University School of Medicine, Baltimore, MD, USA) was used for splicing and quality control (by filtering low-quality sequences) and high-quality sequences were obtained for analysis and processing. The Sanger biocloud information platform (www.majorbio.com (accessed on 1 August 2009) Shanghai Majorbio Technology Co., Ltd., Shanghai, China) was used to analyse the bacterial diversity of sequencing data.

### 2.8. Statistical Analysis

SPSS17.0 software (SPAA Inc., Chicago, IL, USA) was used for variance analysis. A value of *p* < 0.05 represented a significant difference; *p* < 0.01 represented an extremely significant difference. Line charts and bar charts were constructed in Origin 9.0 (OriginLab Corporation, Northampton, MA, USA). Based on the flavour indices of low-salt hotpot sauce, multivariate statistical analysis was performed (16-row and 19-column data matrix). Principal component analysis (PCA), cluster analysis (CA), and multivariate analysis of variance (MANOVA) were used to analyse differences in overall structure and flavour quality among samples. Redundancy analysis (RDA) was then performed to analyse indices significantly related to differences in the overall flavour structure of low-salt hotpot sauce using R ×64 4.1.0 software (Ross Ihaka and Robert Gentleman, University of Auckland New Zealand, Auckland, New Zealand).

## 3. Results and Discussion

### 3.1. Influence of Salt Concentration on Peroxide and Acid Values

**Figure 2:**
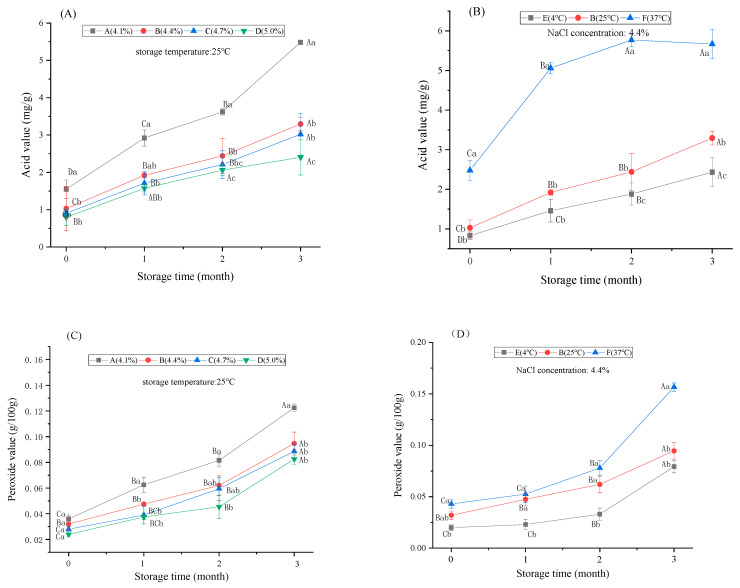
The influence of salt concentration and storage temperature on the AV and POV of hotpot sauce. (**A**,**C**) Comparison of AV (**A**), POV (**C**) of hotpot sauce with different salt concentrations during storage; (**B**,**D**) Effects of different storage temperatures on AV (**B**) and POV (**D**) of hotpot sauce. A, B, C, and D represent significant differences in storage time. a, b, and c represent significant differences between the different groups.

The AV reflects the content of fatty acid in sauce, while the POV directly reflects the degree of rancidity of oil. According to the standard for Chinese hotpot sauce, the AV should be <4 mg/g and the POV should be <0.25 g/100 g. The influence of the salt concentration on the AV and POV of hotpot sauce is shown in Figure 2. At 25 °C, the AV and POV under different salt concentrations increased significantly with storage time, indicating that the oil gradually hydrolysed, leading to rancidity. The AVs of samples with a salt concentration of 4.1% were significantly higher than those of the other groups and the difference became more obvious over time. After being stored at 25 °C for 3 months, the AV of the sauce with a 4.1% salt concentration increased from 1.548 ± 0.246 mg/g to 5.483 ± 0.006 mg/g, exceeding the national standard limit. The AV of other salt concentration groups showed a slow increase trend with the extension of storage time and the higher the salt concentration, the lower the AV. The AVs of 4.4%, 4.7% and 5.0% groups after 3 months of storage increased by 2.266, 2.114, and 1.649 mg/g, respectively, indicating that the increase in salt concentration could slow down the growth of the AV and reduce the production of fatty acids after lipid hydrolysis. The variation trend of the POVs in different salt concentration groups was similar to that of the AVs. The POV of the 4.1% group showed the fastest and most obvious increase from 0.036 ± 0.006 g/100 g to 0.123 ± 0.008 g/100 g. The sauce with a salt concentration of 5.0% deteriorated the most slowly: the AV and POV were 2.404 ± 0.474 mg/g and 0.082 ± 0.008 g/100 g after 3 months of storage.

The influence of the storage temperature on the quality of the sauces was further investigated. The AV and POV also increased significantly with the extension of storage time when the sauce with a 4.4% salt concentration was stored at 4 °C, 25 °C, and 37 °C. It was found that the higher the storage temperature, the more obvious the increasing trend of the AV and POV. The AV of the samples stored at 37 °C for 1 month increased from 2.480 ± 0.250 mg/g to 5.060 ± 0.140 mg/g, exceeding the national standard. After 3 months of storage, the AV reached 5.669 ± 0.372 mg/g. The AV of the 37 °C, 25 °C, and 4 °C storage groups increased by 3.189, 2.266, and 1.603 mg/g after 3 months, respectively, indicating that the lower the storage temperature, the more slower the decomposition of oil and production of fatty acids. Special attention should be paid to the adverse effects of high temperature on hotpot sauces. The AV of the 25 °C and 4 °C storage groups increased slowly and not only met the standard but also maintained good quality within 3 months. The AV of the samples stored at 25 °C and 4 °C for 3 months also maintained 3.294 ± 0.172 and 2.432 ± 0.368 mg/g. The variation trend of the POV was consistent with the AV at different storage temperatures. The 37 °C group showed the most obvious and rapid increase from 0.043 ± 0.004 g/100 g to 0.157 ± 0.004 g/100 g. The POVs of 37 °C, 25 °C, and 4 °C storage groups after 3 months were 0.157 ± 0.004, 0.094 ± 0.008, 0.079 ± 0.006 g/100 g, increased by 0.114, 0.062, and 0.059 g/100 g, respectively. This indicated that low temperature storage is more effective in inhibiting fat oxidation and rancidity. Therefore, the storage temperature is also an important factor affecting the quality of hotpot sauce. In terms of the POV and AV aspects, sauce with a salt concentration above 4.4 is in line with the national standards.

### 3.2. Influence of Salt Concentration on the Flavour of Hotpot Sauce

**Figure 3:**
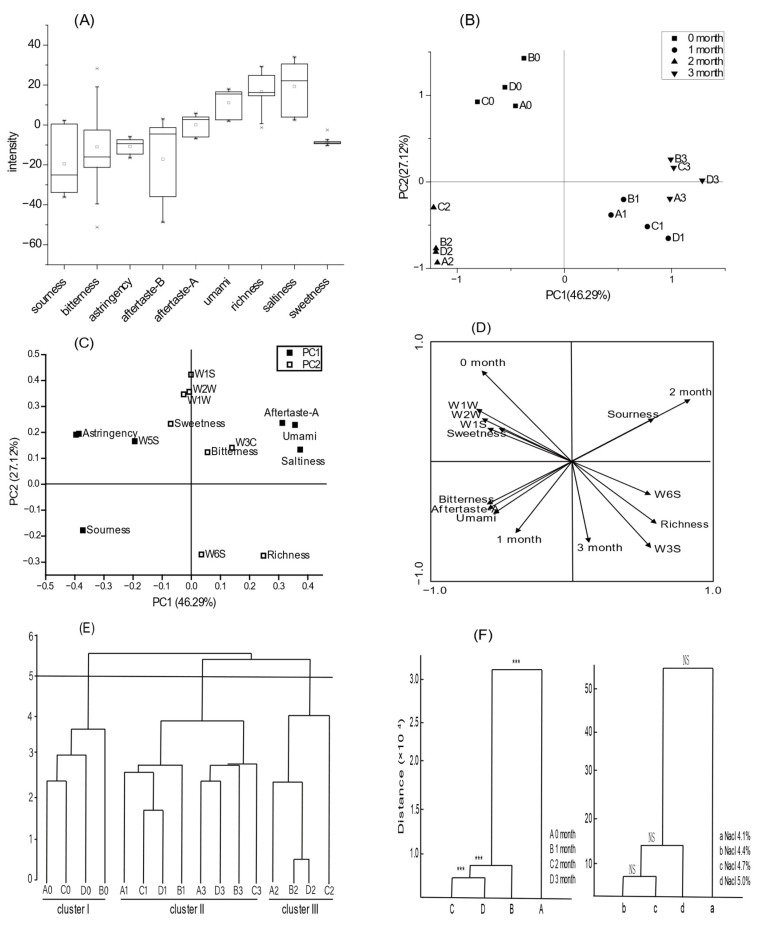
Comparative analysis on taste of hotpot sauces with different salt concentrations during storage. (**A**) Box plot of taste indicators. (**B**) PCA analysis of samples. (**C**) Factor score plot of samples. (**D**) Diplot of the RDA. (**E**) Cluster analysis on the sample quality. (**F**) Cluster analysis of sample quality with different storage time (**left**) and salt concentration (**right**). Note: In (**A**), “*” represented outliers, small square represented the average value. In (**B**,**E**), A–D indicated salt concentration of 4.1%, 4.4%, 4.7%, and 5.0%; 0–3 indicated storage time of 0, 1, 2, and 3 month. In (**F**), “***” represented significant difference.

The electronic tongue was used to analyse the taste of 16 sauce samples with four different salt concentrations during storage and the relative strengths of each taste are shown in Figure 3. Considering that the physicochemical indexes of hotpot sauce do not meet the national standards when stored at 37 °C for 1 month, the overall quality of the samples stored at 4 °C is the best. Therefore, the influence of the salt concentration and storage time on the flavour of hotpot sauce was investigated at a 4 °C storage temperature. The hotpot sauce samples showed the greatest difference in aftertaste B (bitter aftertaste) and sour taste, followed by salty taste, bitter taste, umami taste, aftertaste A (astringent aftertaste), abundance (fresh aftertaste), and astringency, while there was little difference in sweetness (Figure 3A). When the intensity difference of a taste index between two samples was greater than 1, the difference could also be distinguished in terms of sensory quality. The mean differences in six basic taste indices and three aftertaste indices were greater than 1 (38.05, 7.24, 10.51, 51.60, 12.29, 16.03, 30.5, 31.48, and 7.79, respectively). Thus, the differences in the above indices of hotpot sauce quality could be distinguished by taste identification, i.e., there were marked differences in the taste of hotpot sauce samples with different salt concentrations.

Xie et al. [19] also studied the influence of added salt (12–21%) on the sensory quality of shrimp sauce and reported that the sauce with appropriate salt levels (15% and 18%) had a strong aroma, harmonious flavour, delicate taste, and the best overall acceptance. During the post-ripening process, too little salt is not conducive to the formation of colour and aroma and affects the moisture content of the product, thus reducing the viscosity. However, excessive salt not only leads to salty products but also inhibits protease activity, causing incomplete protein hydrolysis and reducing the production of amino acids, thus reducing the umami taste.

### 3.3. Influence of Salt Concentration on the Volatile Flavour Quality of Hotpot Sauce

An electronic nose was used to evaluate the volatile flavour compounds of 16 samples with different salt concentrations during storage. The differences in the relative intensities of each flavour indicator are shown in Table 1. The 10 sensors showed different response values; the values for sensors W5S, W6S, W1S, W1W, W2S, W2W, and W3S were relatively high, ranging from 1.0 to 4.1, while those of the other three sensors were all less than 1.0. The results showed that the contents of nitrogen oxides, hydrogen, methane, organic sulphides, terpenoids, ethanol, and alkanes increased in the samples during storage and nitrogen oxides had the highest response values.

The main ingredients of the hotpot sauce were peanuts and sesame seeds. Xu et al. [20] reported that the main flavour substances of sesame paste were aldehydes, alcohols, pyrazines, and hydrocarbons. Wang et al. [21] studied the flavour of peanut butter using an electronic nose and reported that the main flavour substances were methyl groups, nitrogen oxides, sulphides, aromatic components, and organic sulphides, similar to those identified in this study. According to the variation values, the differences in 16 sauce samples were greatest in W1W (organic sulphide and terpenoids); followed by W5S, W2W, W1S, W2S, and W3S; while the differences in W1C, W3C, W5C (which are sensitive to aromatic substances), and W6S were relatively small.

### 3.4. Analysis of the Overall Flavour Structure of Hotpot Sauce

Multivariate statistical methods, such as CA, PCA, and MANOVA, were used to study the overall flavour structure of hotpot sauce. CA was conducted on 16 samples based on Euclidean distance, as shown in Figure 3. With a distance of 5, the 16 samples could be divided into three clusters that showed obvious differences. Four samples belonged to cluster I (A0, B0, C0, and D0), eight to cluster II (A1, B1, C1, D1, A3, B3, C3, and D3), and four to cluster III (A2, B2, C2, and D2) (Figure 3E). The results suggested that the storage time may be the key factor affecting the flavour quality of low-salt hotpot sauce, followed by the salt concentration.

To verify this, CA and MANOVA were conducted to determine the effects of the storage time and salt concentration on flavour quality and the results are shown in Figure 3F. No significant differences were found in the flavour quality between salt concentration clusters (*p* > 0.05), while there were significant differences between storage time clusters (*p* < 0.001). When the distance was about 5 × 10^4^, the samples were divided into four groups according to the salt concentration. This indicated that the storage time is the main factor affecting the flavour quality of low-salt hotpot sauce, consistent with the results shown in Figure 3A.

PCA was then used to analyse the overall differences in quality indices. The information on sample quality was mainly concentrated in the first two principal components (PC), which explained 73.41% of the variance (Figure 3B). The storage time clusters showed obvious spatial separation on PCA-factor loading diagrams. The original samples, and those stored for 1, 2, and 3 months, were located in the second, first, third, and fourth quadrants, respectively. The 1- and 3-month groups were relatively concentrated and clearly differentiated from the other two groups, which is consistent with the results shown in Figure 3A,B. The PCA-factor loading diagrams are shown in Figure 3C. The first PC (PC1) was composed of seven indices, i.e., saltiness, umami, aftertaste A, sour taste, astringency taste, aftertaste B, and W5S, together accounting for 46.29% of the variance. The second PC (PC2) was composed of eight indices, i.e., W1S, W2W, W1W, W3C, W6S, sweet, bitter, and abundance, together accounting for 27.12% of the total variance. Thus, PC1 was mainly composed of taste indices of low-salt hotpot sauce, while PC2 was composed of volatile flavour and taste indices.

As shown in the factor score plot, in the x-axis direction, the freshness, salty taste, and taste A (astringent aftertaste) of low-salt hotpot sauce first showed increasing trends with the storage time; sour, astringency, and aftertaste B (bitter aftertaste) then increased along with nitrogen oxide content, which may have been influenced by the fat hydrolysis of the peanut kernels, sesame, and soybeans, as well as the microbial activities of leek flower. Finally, umami taste, salty taste, and aftertaste A showed increasing trends. In the direction of the y-axis, the response values of sensors W1S (sensitive to methane), W1W (sensitive to organic sulphide and terpenoids), W2W (sensitive to organic sulphide), W3C (sensitive to ammonia and aromatic substances), sweet and bitter taste first decreased and then increased with storage time. W6S (sensitive to hydrogen) and abundance (fresh aftertaste) increased in the middle stage of storage. These results may have been due to the stability of the sugar and sulphur compounds, such as allicin in garlic, which are not volatile, leading to their gradual emergence during the storage process.

RDA was further used to identify and analyse specific factors responsible for the differences in the overall flavour structure, as shown in Figure 3D. Six taste indices, i.e., sweet, sour, bitter, umami, aftertaste A (bitter aftertaste), and abundance (fresh aftertaste) and five volatile flavour indices, i.e., W1W, W2W, W1S, W3S, and W6S, showed good correlations. The chemical substances represented by the above indicators were the main taste and flavour components responsible for the overall quality differences in low-salt hotpot sauce according to storage time. W1W, W2W, W1S, and sweet were associated most with the original sample side; bitter, aftertaste A, and umami were associated most with the 1-month group; sour was associated most with the 2-month group; and W6S, W3S, and abundance were associated most with the 3-month group. These results showed that the quality indices of low-salt hotpot sauce, i.e., bitter taste, astringent aftertaste, sour taste, and W3S, were the most prominent and the quality decreased with storage time.

### 3.5. Bacterial Diversity Analysis of Low-Salt Hotpot Sauce

The bacterial diversity of the samples with different salt concentrations stored at 4 °C and 25 °C for 3 months was examined. A total of 469,026 valid sequences were obtained from the eight samples. Based on a classification cut-off of 97% similarity, a total of 440 operational taxonomic units (OTUs) were annotated and the sequencing coverage was >99.97%. The dilution curves of the Sobs and Shannon indices of each sample tended to be gentle, indicating that the amount of sequencing data was reasonable and reflected most of the bacterial community diversity information in the samples (Figure S1).

**Figure 4:**
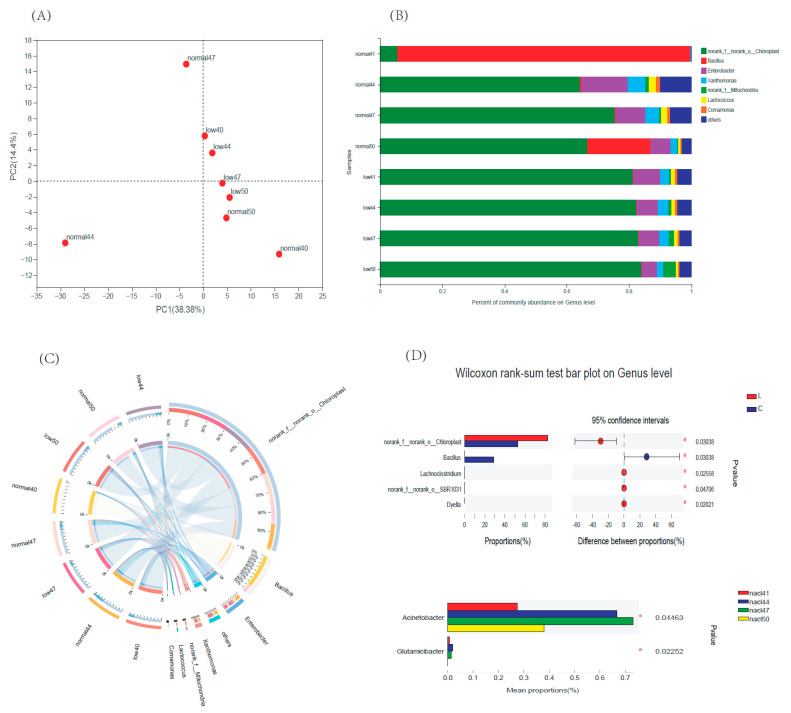
Comparative analysis on the bacteria composition of hotpot sauces with different salt concentrations. (**A**) PCA analysis of samples. (**B**) Bacteria composition on the genus level. (**C**) Circos plot of bacterial composition comparison. (**D**) Wilcoxon rank-sum test bar plot on genus level. Note: “*“ indicated *p* ≤ 0.05.

There were obvious differences in bacterial compositions between the low- and normal-temperature samples. On PCA, the low-temperature samples were clustered with 5% salt samples at normal temperatures and were distant from other sample groups, indicating that both low temperatures and high amounts of salt had inhibitory effects on miscellaneous bacteria, resulting in a similarity in the composition of bacterial communities between the two groups (Figure 4A). A total of 440 OTUs belonging to 247 genera and 21 phyla were identified in all the samples. At the phylum level (Figure S2), there were no significant differences in bacterial composition among low-temperature samples. Cyanobacteria were the dominant phylum, followed by Proteobacteria and Firmicutes. In contrast, the bacterial composition of the samples stored at room temperature differed significantly according to the salt concentration. The dominant phylum of the 4% salt group was Firmicutes. With an increase in the salt concentration, Cyanobacteria and Proteobacteria became the dominant phyla of the hotpot sauce and the bacterial community structure gradually approached that of the samples stored at low temperatures. In addition, the amount of Firmicutes in the samples increased significantly at the high-salt concentration of 5%.

At the genus level (Figure 4B,C), *norank-f-o-Chloroplast* (Cyanobacteria) was the main bacterial genus in the samples stored at low temperatures, followed by *Enterobacter*, *Xanthomonas*, *norank-f-Mitochondria*, and *Lactococcus*. At room temperature, 4% salt-concentration samples showed high *Bacillus* content, indicating that storing low-salt sauce without preservatives at room temperature is unsafe. Increasing the salt concentration had an obvious inhibitory effect on the *Bacillus* content. However, it is not sufficient to rely on the salt concentration alone to inhibit these bacteria because *Bacillus* was also detected in 5% salt samples at room temperature, indicating that it is necessary to use additional preservatives, such as Nisin, to inhibit contamination by *Bacillus* and other miscellaneous bacteria during the production of hotpot sauce. Similarly, the amount of salt added also affected the total number of bacteria in low-salt shrimp sauce [19]. The total number of bacteria showed an increasing trend with decreasing amounts of salt added, indicating that, under the same sterilization conditions, the salt concentration decreased significantly and the bacteriostatic effect significantly weakened.

A Wilcoxon’s rank-sum test was performed to examine the differences in bacterial genera among the different groups. Five genera showed significant differences between storage at 4 °C and 25 °C, i.e., *norank-f-o-Chloroplast*, *Lachnoclostridium*, *norank_f-norank-o-SBR1031*, *Dyella*, and *Bacillus*. The contents of *norank-f-o-Chloroplast*, *Lachnoclostridium*, *norank-f-norank-o-SBR1031*, and *Dyella* at low temperatures were significantly higher than those at 25 °C. The results showed that cold storage allowed the growth of *norank-f-o-Chloroplast* and three other bacterial genera and can properly inhibit the production of bacillus, but in the storage temperature above 25 °C, the breeding of bacillus should be careful [22]. The same phenomenon also occurred in Chinese spiced beef. The total number of colonies exceeded the limit standard when stored at 4 °C for 6 days and at 25 °C for 2 days, including pseudomonas, staphylococcus, lactic acid bacteria, and bacillus. The shelf life was 5.83d at 4 °C and 1.69d at 25 °C [23].

Zheng et al. [24] detected spoilage microorganisms in hotpot sauce products with dilated bags. Morphological examination, physiological and biochemical analyses, 16S rDNA sequence identification, and phylogenetic tree analysis identified three spoilage microorganisms: *Bacillus amyloliquefaciens*, *Bacillus licheniformis*, and *Bacillus subtilis*. *Bacillus* is an anaerobic microorganism with strong stress resistance, low nutritional requirements, a high recurrence rate, a rapid growth rate, the ability to produce a variety of enzymes, and strong heat resistance. High *Bacillus* content at a late stage might hinder sterilization. Due to the complex ingredients in the hotpot sauce, several auxiliary materials were added during processing, which could introduce different bacteria. The method of sterilization at 80 °C for 10–20 min can only kill most yeast and mould because of uneven heat transfer; some high-temperature-resistant spores can still survive, resulting in spoilage.

Different salt concentrations also led to different bacterial communities in hotpot sauces, with the main differential bacterial genera being *Acinetobacter* and *Glutamicibacter*. With an increasing salt concentration, the contents of these two bacterial genera first increased and then decreased, indicating that a high salt concentration can indeed have a bacteriostatic effect and that a low-salt-concentration sauce should be combined with preservatives to ensure safety.

### 3.6. Correlations between Microflora and Flavour Quality

**Figure 5:**
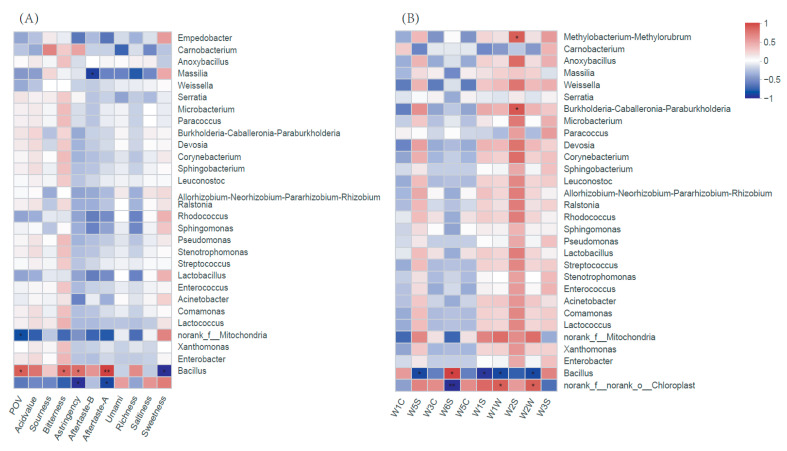
Correlation analysis on the physicochemical, taste (**A**), flavour indices (**B**), and bacterial composition of hotpot sauce. Note: “*“ indicated *p* ≤ 0.05, “**” indicated 0.001 ≤ *p* ≤ 0.01.

A correlation analysis was conducted on the physicochemical, taste, and flavour indices of hotpot sauce (Figure 5). As shown in Figure 5A, *Bacillus* was positively correlated with POV and AV, indicating that it directly promoted the spoilage of hotpot sauce. *Bacillus* was also significantly positively correlated with the bitter taste, astringent taste, and astringent aftertaste of hotpot sauce and negatively correlated with a sweet taste, indicating that it was the main bacterial genus responsible for the deterioration in the taste of spoiled hotpot sauce. *Bacillus* was negatively correlated with the response values of the seven sensors (Figure 5B), suggesting that *Bacillus* may be involved in the decomposition of most volatile flavour compounds, including nitrogen oxides, aromatic compounds, alkanes, and sulphides. *Bacillus* causes undesirable flavours in many foods, such as *Alicyclobacillus acidoterrestris* and *Alicyclobacillus acidocaldarius*, which can cause spoilage and produce an unacceptable flavour during pasteurized juice production. In the early stages of spoilage, there was no obvious swelling or rancidity, but a relative concentration of the metabolites of this bacterium of one trillionth would adversely affect the taste and flavour of the juice, resulting in turbidity and even white precipitate and other quality-reducing effects.

*Norank-f-norank-o-Chloroplast* was negatively correlated with POV and AV, as well as with sourness, bitterness, astringency, aftertaste B, and aftertaste A. Thus, the presence of *norank-f-norank-o-Chloroplast* was beneficial for the preservation and flavour of hotpot sauce. In addition, *norank-f-norank-o-Chloroplast* was positively correlated with nitrogen oxides, aromatic compounds, alkanes, sulphides, and other volatile flavour substances, suggesting that the genus *norank-f-norank-o-Chloroplast* was beneficial for the formation of flavour compounds. Bacterial genera other than *Bacillus* and *Carnobacterium* were positively correlated with the response values of the W2S sensor, suggesting that most of the bacterial genera were related to the formation of alcohols, especially *Methylobacterium*-*Methylorubrum* and *Burkholderia*-*Caballeronia*-*Paraburkholderia*

## 4. Conclusions

In this study, changes in the physicochemical quality, flavour, and bacterial diversity of hotpot sauces with different salt concentrations were investigated during storage at different temperatures and correlation analysis was carried out. The results showed that the quality of hotpot sauce deteriorated gradually with storage and that storage under 4 °C and salt-concentration above 4.4% conditions could significantly reduce spoilage. The salt concentration had no significant effect on the flavour of hotpot sauce, but the flavour quality decreased significantly with storage time. *Norank-f-o-Chloroplast* was the main bacterial genus under cold storage (4 °C) conditions, which was beneficial for the preservation of sauce and the improvement in flavour. However, *Bacillus* was detected in 4% salt samples stored at 25 °C, directly promoted the spoilage of the sauce, and was significantly related to the deterioration of the sauce flavour. This bacterium signalled the spoilage of low-salt hotpot sauce stored at room temperature. Therefore, low-salt hotpot sauce requires preservatives and a cold storage environment to ensure it is safe for consumption.

## Figures and Tables

**Table 1:** The analysis of relative intensity of each flavour index in low-salt hotpot sauce samples stored in 4 °C (*n* = 16, 4 different concentration × 4 month).

Sensors	Performance Description	Mean Value	Median	Minimum	Maximum	Range	Deviation Value/%
W1C	Sensitive to aromatic substances	0.78	0.80	0.70	0.83	0.13	5.61
W5S	Sensitive to nitrogen oxides	3.56	3.58	1.06	6.70	5.64	46.54
W3C	Sensitive to ammonia and aromatic substances	0.96	0.95	0.92	0.99	0.064	2.09
W6S	Selective for hydrogen	1.18	1.18	1.09	1.30	0.22	4.72
W5C	Sensitive to alkane and aromatic substances	0.98	0.98	0.96	1.01	0.04	1.24
W1S	Sensitive to methane	2.89	3.02	1.97	4.32	2.35	26.68
W1W	Sensitive to organic sulfides and terpenes	2.51	2.28	1.14	6.60	5.47	59.03
W2S	Sensitive to ethanol	2.65	2.37	1.98	3.82	1.83	24.66
W2W	Sensitive to organic sulfides	1.69	1.64	1.08	3.10	2.02	33.14
W3S	Sensitive to alkanes	1.58	1.56	1.27	1.87	0.60	11.20

## Data Availability

Data is contained within the article and Supplementary Materials.

## Supplementary Materials

The following supporting information can be downloaded at: https://www.mdpi.com/article/10.3390/foods12020333/s1, Figure S1: The dilution curves of the Sobs and Shannon indices of hotpot sauces with different salt concentrations during storage. Figure S2: Comparative analysis on the bacteria composition of hotpot sauces with different salt concentrations on phylum level.
