# Supplementary material for: Variation and Correlation Analysis of Flavour and Bacterial Diversity of Low-Salt Hotpot Sauce during Storage
Source: Foods. 2023 Jan 10;12(2):333. doi: 10.3390/foods12020333 (PMC9857581; doi:10.3390/foods12020333)
Supplement: Supplementary file 1 [file foods-12-00333-s001.zip › foods-2052076-supplementary.pdf]

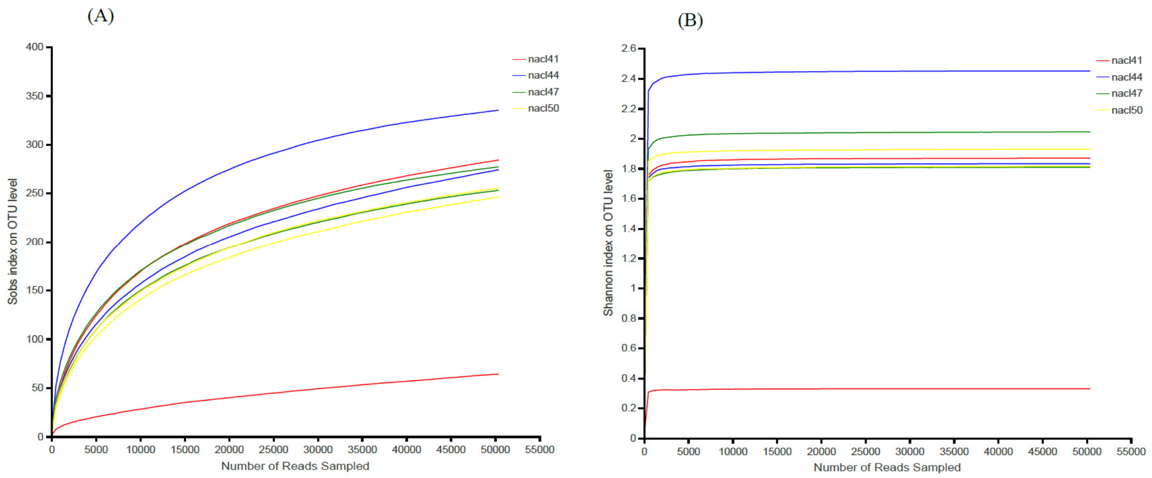

Figure S1 The dilution curves of the Sobs and Shannon indices of hotpot sauces with different salt concentrations during storage.

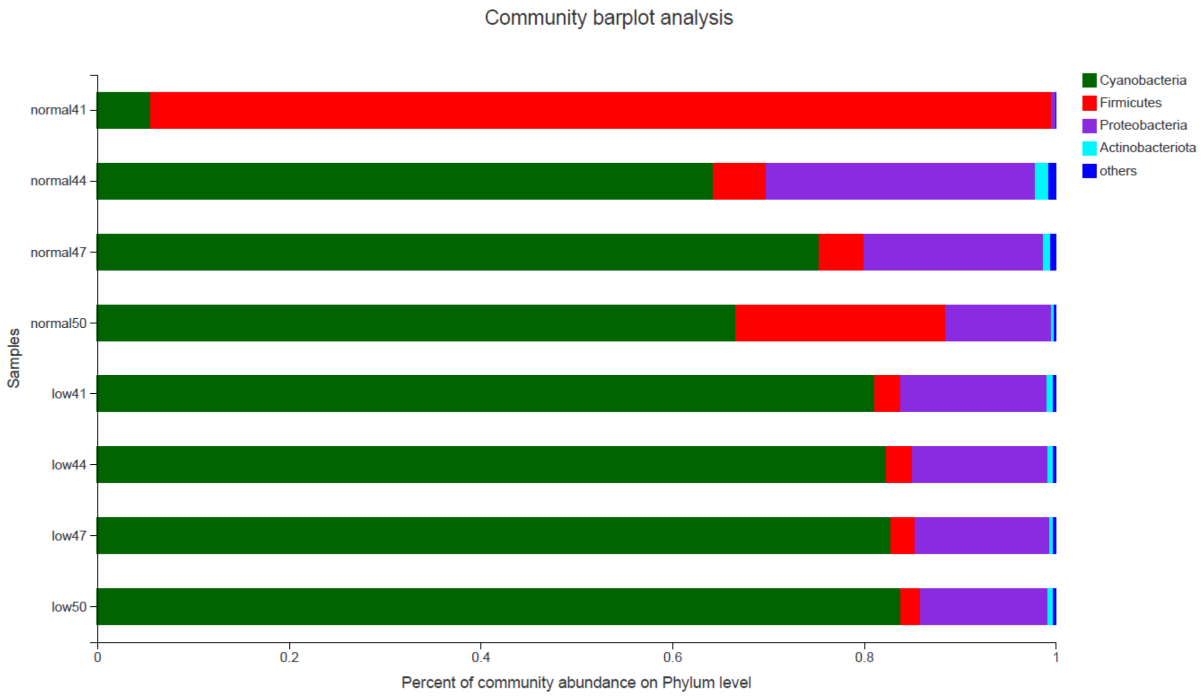

Figure S2 Comparative analysis on the bacteria composition of hotpot sauces with different salt concentrations on phylum level
